# Usability, Acceptability, and Preliminary Effectiveness of a Peer-Delivered and Technology-Supported Mental Health Intervention for Family Caregivers of People With Dementia: Field Usability Study

**DOI:** 10.2196/41202

**Published:** 2024-05-27

**Authors:** Caroline Collins-Pisano, Amanda N Leggett, David Gambee, Karen L Fortuna

**Affiliations:** 1 Department of Psychological and Brain Sciences Dartmouth College Hanover, NH United States; 2 Department of Psychology University of Colorado, Colorado Springs Colorado Springs, CO United States; 3 Institute of Gerontology Wayne State University Detroit, MI United States; 4 Department of Psychiatry Dartmouth College Hanover, NH United States

**Keywords:** family caregivers, dementia, peer support, technology, mobile phone

## Abstract

**Background:**

Family caregivers of people with dementia are critical to the quality of life of care recipients and the sustainability of health care systems but face an increased risk of emotional distress and negative physical and mental health outcomes.

**Objective:**

The purpose of this study was to examine the usability, acceptability, and preliminary effectiveness of a technology-based and caregiver-delivered peer support program, the Caregiver Remote Education and Support (CARES) smartphone or tablet app.

**Methods:**

A total of 9 adult family caregivers of people with dementia received the CARES intervention, and 3 former family caregivers of people with dementia were trained to deliver it. Quantitative data were collected at baseline and at the end of the 2-week field usability study. Qualitative data were also collected at the end of the 2-week field usability study.

**Results:**

The field usability study demonstrated that a 2-week peer-delivered and technology-supported mental health intervention designed to improve burden, stress, and strain levels was experienced by former and current family caregivers of people with dementia as acceptable. Current family caregivers rated CARES as above average in usability, whereas the caregiver peer supporters rated CARES as marginally usable. CARES was associated with non–statistically significant improvements in burden, stress, and strain levels.

**Conclusions:**

This field usability study demonstrated that it is possible to train former family caregivers of people with dementia to use technology to deliver a mental health intervention to current family caregivers of people with dementia. Future studies would benefit from a longer trial; a larger sample size; a randomized controlled design; and a control of covariables such as stages of dementia, years providing care, and severity of dementia symptoms.

## Introduction

### Background

Family caregivers of people with dementia are critical to the quality of life of care recipients and to the sustainability of health care systems. Family caregivers provide US $257 billion in unpaid care to people living with dementia but face an increased risk of emotional distress and negative physical (eg, heart disease and hypertension) and mental (eg, depression and anxiety) health outcomes [1,2]. While positive gains are reported in the caregiving role, caregivers are more likely than their noncaregiving peers to report stress, burden, and strain. Approximately 46% of family caregivers of people with dementia are classified as having high levels of burden [1,2]. Burden is defined as the psychological, physical, emotional, and social challenges that family caregivers experience in response to the demands of providing care [3]. Caregivers with high levels of burden report more physical and psychological symptoms, use prescription medications and health care more frequently, and provide poorer quality of care to recipients, leading to an increased likelihood of premature institutional care [4,5]. In addition, 59% of family caregivers of people with dementia rate the emotional stress of caregiving as high or very high, and 38% rate the physical stress of caregiving as high or very high [1,2]. Stress is defined as individuals’ emotional or physical responses to the challenges of caregiving, such as fatigue [6,7]. The stress of providing care to a family member with dementia increases caregivers’ risk of health complications, increases their susceptibility to diseases such as hypertension, and negatively affects their quality of sleep [1,2]. Family caregivers have also reported greater levels of strain compared to caregivers of people without dementia. Strain is defined as caregivers’ perception of the challenges of caregiving and their state of well-being [7]. Family caregivers who perceived themselves as having higher strain levels due to caregiving responsibilities were at a higher risk of death than those who perceived little or no strain [1,2].

### Technology-Based Interventions for Caregivers

While psychosocial interventions have been shown in fully powered randomized controlled trials to reduce caregiver burden and delay nursing home admission for the care recipient, a recent meta-analysis by Walter and Pinquart [8] found that current interventions had a disappointingly small to moderate effect on caregiver well-being, burden, depression, and anxiety [4,8-10]. In addition, uptake of these interventions in the real world is limited due to caregiving obligations (between 69 and 117 hours of informal care are provided to people with dementia per week), geographical distance from the intervention, requirements to meet in person, and failure to address the personalized needs of the family caregivers.

Technology-based interventions may offset these challenges through improved accessibility of psychosocial interventions at any time or location [11,12]. A scoping review on existing technology-based peer support interventions for family caregivers found that web-based programs include websites that offer educational materials with the option to contact other informal caregivers, web-based portals with psychoeducation and peer-to-peer contact, asynchronous e-learning platforms, internet support forums and chat rooms, videoconferencing support groups facilitated by a health professional, and live virtual reality support groups facilitated by psychologists [11]. Online informal peer support groups for informal caregivers have shown high levels of engagement. Technology-based interventions enable caregivers to participate even if they are unable to leave the person with dementia unsupervised. For example, during the COVID-19 pandemic, telephone-, videoconference-, and chat room–based online support groups were the only media accessible to caregivers, a benefit due to the 24/7 nature of supporting a family member with dementia and the consequential challenges in accessing support [11,13]. Online informal peer support groups are an effective method of asynchronous web-based delivery when offered in combination with a structured psychosocial and educational intervention by skilled clinical professionals [11]. However, while some studies such as that by Han et al [14] have shown significant reductions in depression, stress, and helplessness, others such as the study by Marziali and Garcia [15] have shown only moderate effects in reducing burden and depression and increasing caregiver knowledge.

### Significance of Technology-Delivered Caregiver Peer Support

To date, technology-based interventions have relied on a skilled workforce of geriatric mental health professionals. However, there are insufficient numbers of adequately trained geriatric mental health care providers [16]. As such, task shifting services from skilled clinical professionals to community members provides an emerging workforce of peer support workers (ie, interventionists without formal mental health education but with life experiences similar to those of the people they serve) [17]. Although the need for traditional clinical professionals remains, peer support services for individuals with mental health conditions have been shown to increase service accessibility without impacting service quality [17]. However, there is limited knowledge of caregiver-delivered peer support.

The use of caregiver-delivered digital peer support may promote the uptake of psychosocial interventions, reduce burden, and delay nursing home admission. Social and behavioral theories such as social support, experiential knowledge, and the helper therapy principle highlight how peer supporters have the unique ability to offer acceptance, understanding, and validation to the individuals they work with [18]. Because of their shared lived experiences, peers are often viewed as more credible and trustworthy than other health care providers and, therefore, encourage increased digital health engagement. Individuals are motivated to achieve their mental health goals (eg, reductions in burden, strain, and stress) because of the reciprocal accountability offered and modeled by their peers. Former family caregivers of people with dementia have the knowledge and skillset to deliver trained peer support to current family caregivers and could potentially benefit emotionally from the delivery of support. While there are positive outcomes associated with the end of caregiving, when family caregivers of people with dementia become former family caregivers, the detrimental effects of previous caregiving fail to improve [19]. Many former dementia caregivers experience persistent sleep disturbances, depression, anxiety, increased physician office visits, declining health, and feelings of guilt and regret [20]. Despite evidence that former caregivers who pursue new caring roles benefit emotionally, payers and health care providers have not hired and trained former caregivers to provide evidence-based digital interventions [20].

### Research Aims

The purpose of this study was to examine the usability, acceptability, and preliminary effectiveness of a technology-based and caregiver-delivered peer support program, the Caregiver Remote Education and Support (CARES) smartphone or tablet app. In this study, 3 former family caregivers of people with dementia (caregiver peer supporters) were trained in the delivery of caregiver peer support and delivered the CARES intervention and app to 9 current family caregivers of people with dementia (caregiver participants) in a 2-week field usability study.

### Design of the CARES App

The CARES app and intervention were adapted from the PeerTECH smartphone or tablet app and developed to facilitate the examination of the usability, acceptability, and preliminary effectiveness of the first technology-based and caregiver-delivered peer support program in a 2-week field usability study. The PeerTECH system was designed using universal design principles and for lay interventionists (not skilled) to deliver fidelity-adherent evidence-based interventions. The PeerTECH system has been successfully used with Certified Peer Specialists, home health aides, and Certified Older Adult Peer Specialists [21]. Certified Peer Specialists are people with a mental health diagnosis who are hired, trained, and certified to provide peer support services to individuals with a similar diagnosis [22].

PeerTECH was built on the stress-vulnerability model. According to the stress-vulnerability model, the outcomes of a mental health condition are connected to biological vulnerability, stress, and protective factors (eg, peer support) [23]. PeerTECH was designed to empower individuals to address the stress and vulnerability that lead to worsening medical, psychiatric, and social health conditions. Peer specialists are trained to deliver evidence-based practices through the PeerTECH app to help participants decrease stressors and increase protective factors.

The PeerTECH app was codeveloped with peer specialists and includes 2 features developed through community-engaged research. The first is a peer-facing smartphone or tablet app that guides peers in delivering evidence-based health self-management skill development interventions. The app includes prompts to share their lived experiences of health challenges and solutions (ie, peer support) and structured intervention delivery through scripted, evidence-based training on topics such as coping skills, psychoeducation, medical management, social skills, and self-advocacy. The second feature is a participant-facing app that offers self-management support through features such as a library of self-management resources (eg, peer-led videos) and a secure messaging feature to connect with their assigned peer specialists and reinforce goals [21].

**Figure 1:**
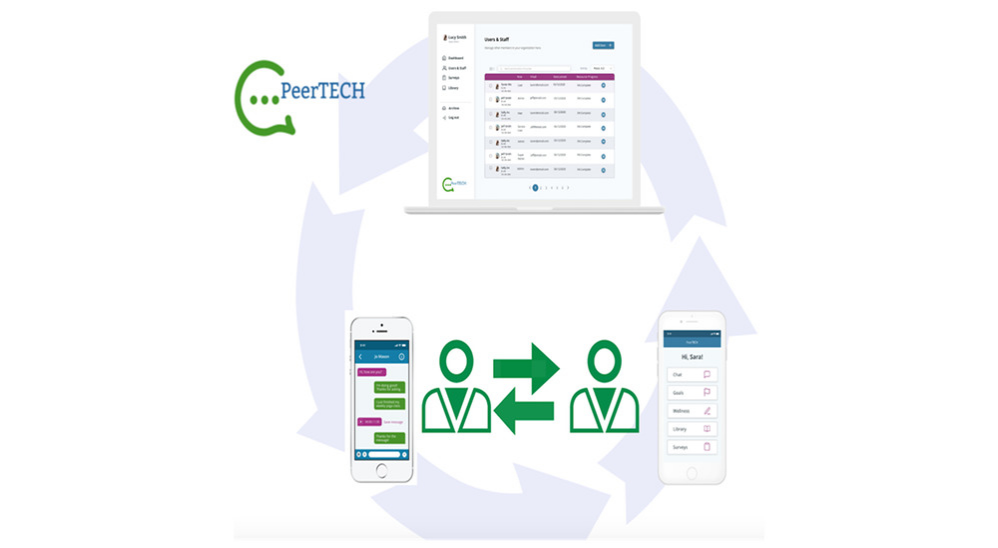
Caregiver Remote Education and Support app.

Similar to PeerTECH, the CARES app includes 2 features: a peer-facing smartphone or tablet app and a participant-facing smartphone or tablet app (Multimedia Appendix 1 for illustrations of the CARES app). The content of the CARES app, similar to that of PeerTECH, was designed according to the stress-vulnerability model to help participants decrease stressors and increase protective factors. The CARES app and intervention were adapted from the PeerTECH app by a team of researchers with expertise in peer support to guide caregiver peers in the delivery of evidence-based mental health interventions and designed according to the techniques and principles of peer support and acceptance and commitment therapy (ACT). The participant-facing CARES app offers a library of resources designed to educate participants on psychological skills (eg, mindfulness) that empower them to manage difficult thoughts (eg, acceptance) and engage in activities and behaviors that are guided by their life values and boost their well-being (eg, setting goals and identifying values) [24]. Caregiver peer supporters were trained and educated on topics such as values, goal setting, acceptance, avoidance, and negative thoughts and connected with their assigned participants via a secure messaging feature to reinforce ACT principles, share their lived experience of caregiving challenges, and share practices to enhance caregivers’ wellness and mental health in relation to caregiver-related stressors [25]. The mutual practice of goal setting, for example, can help link caregivers’ values to concrete plans for behavior change [26]. Figure 1 illustrates the CARES app.

The participant-facing app includes access to direct messaging with an assigned caregiver peer supporter, goals, wellness, surveys, and a resource library (see the bottom right of Figure 1). The caregiver peer supporter app allows caregiver peer supporters to message assigned participants directly (see the bottom left of Figure 1), view participants’ goals and wellness plans, and view their progress in the library resource feature. The principal investigator (PI) had access to data on the participants’ and peer supporters’ rate of engagement with library resources, messaging, and goals and wellness features (see the top of Figure 1).

### Description of the CARES App

**Figure 2:**
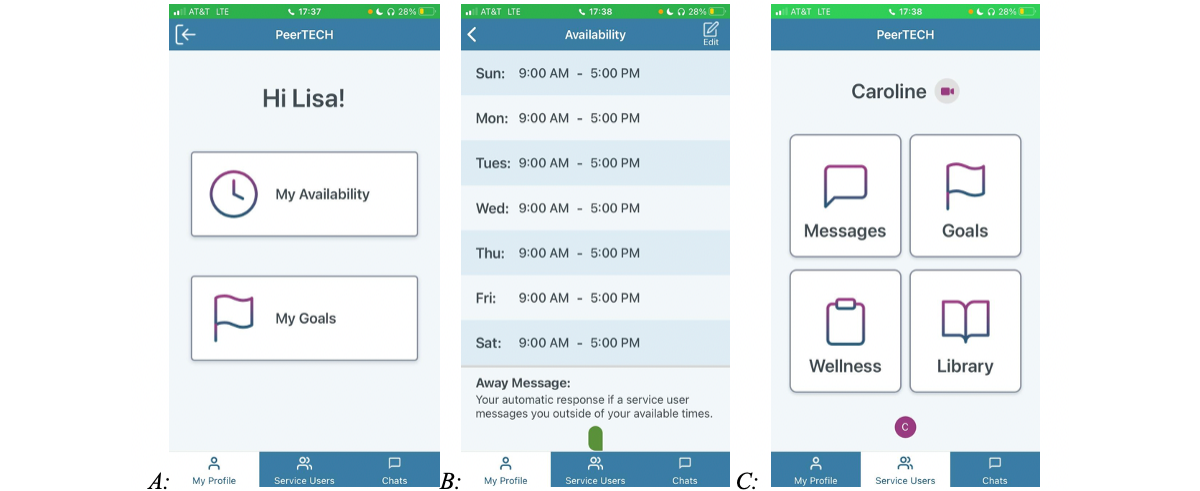
Screenshot A depicts the home page on the caregiver peer supporter facing CARES application. The homepage includes the option to select the individual’s availability to offer caregiver peer support to their assigned participants (see Screenshot B), set goals, and access information on their assigned participants and chats. Screenshot C provides options to message and video chat the assigned participant and track their goals and wellness plan.

**Figure 3:**
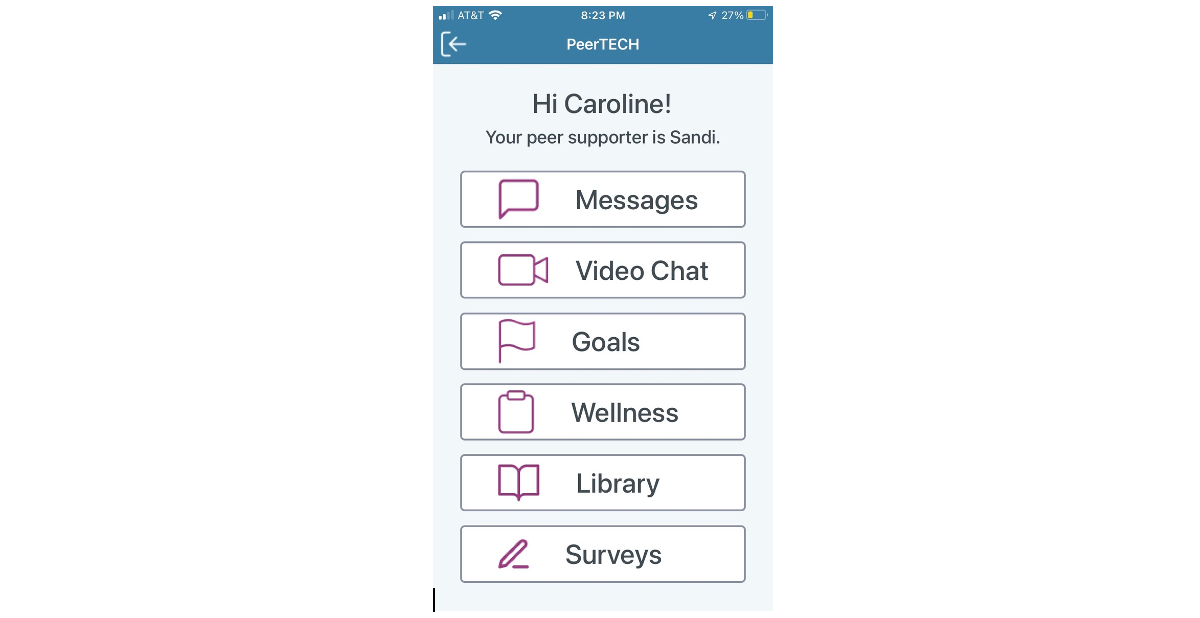
Caregiver Remote Education and Support participant-facing app.

**Figure 4:**
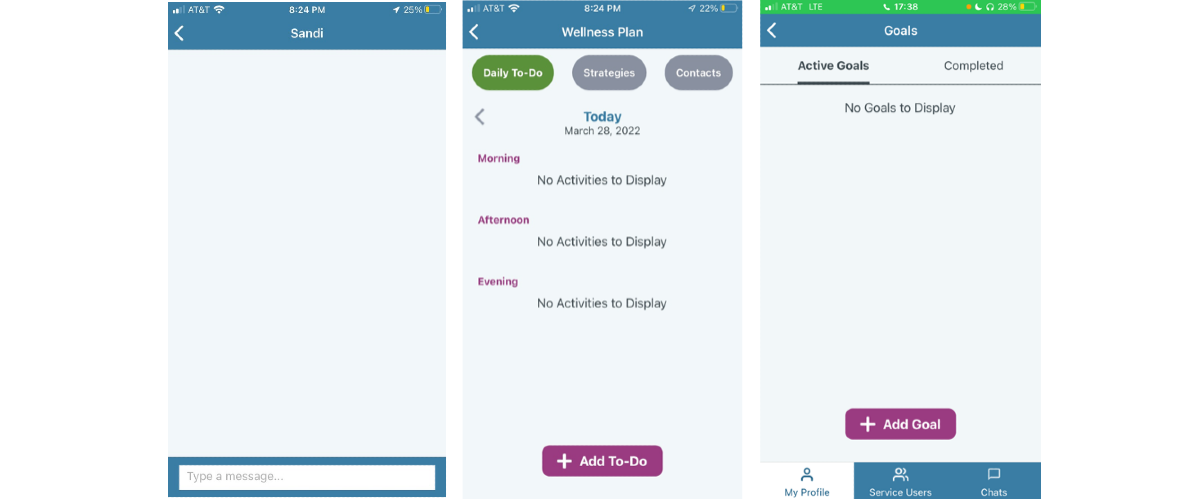
Caregiver Remote Education and Support features on both the participant- and caregiver peer supporter–facing apps.

Fundamentally, CARES is a peer-instructed and mediated caregiver support program that uses a smartphone app–based mechanism for communication. The CARES app consists primarily of two features: (1) a former family caregiver (caregiver peer supporter)–facing app on a smartphone or tablet that includes the option to message or video chat with the current family caregivers (participants) they have been assigned to provide caregiver peer support (Figure 2) and (2) a participant-facing app that offers the option to review an on-demand library of educational resources and a HIPAA (Health Insurance Portability and Accountability Act)-compliant text and video chat feature to communicate with their assigned caregiver peer supporter (Figure 3). The participants and caregiver peer supporters also have the option to set goals and create wellness plans. Figure 4 shows the features seen by both the participants and caregiver peer supporters.

Figure 2A depicts the home page of the caregiver peer supporter–facing CARES app. The home page includes the option to select the individual’s availability to offer caregiver peer support to their assigned participants (Figure 2B), set goals, and access information on their assigned participants and chats. Figure 2C shows the options to message and video chat with the assigned participant and track their goals and wellness plan.

**Figure 5:**
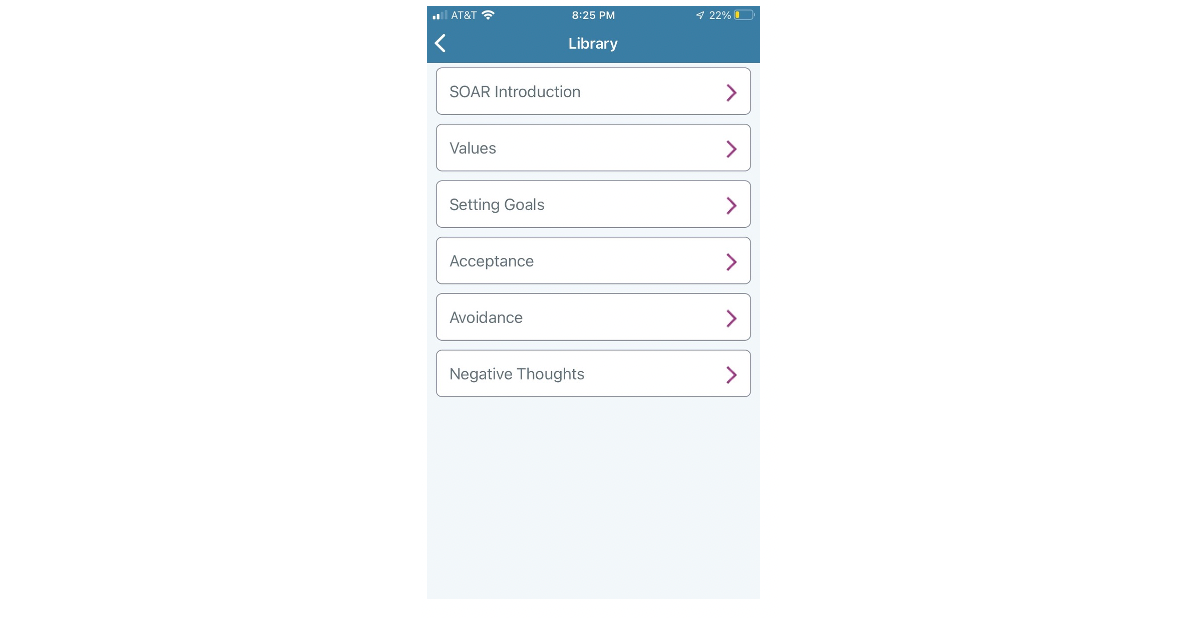
Caregiver Remote Education and Support library of resources.

Figure 3 depicts the home page of the participant-facing CARES app. The home page includes the option to send messages to the assigned caregiver peer supporter, video chat directly with the assigned caregiver peer supporter, set goals, create a wellness plan, and access a library of resources (Figure 5).

The left panel in Figure 4 depicts the messaging option provided within the CARES app. Within the messaging section of the app, participants and their assigned caregiver peer supporters can send each other text-like messages. The panel in the center depicts the wellness plan. Within the wellness plan section, participants and caregiver peer supporters can add activities and strategies they wish to complete to enhance their wellness. The panel on the right depicts the goals section. Within the goals section, participants and caregiver peer supporters can set goals for themselves.

### Description of the CARES Resource Library

The CARES app includes a resource library with materials related to the principles of peer support and evidence-based practices and skills, such as ACT, to manage stress and promote mental health and well-being. CARES educational resources are designed to be reviewed by a participant and caregiver peer supporter together or individually (Figure 5). Each resource includes a combination of videos and text.

Figure 5 depicts the library of resources found in the CARES app. Each topic includes a scripted curriculum with evidence-based practices to improve mental health and well-being.

## Methods

### Overview

A field usability study was conducted in April 2022 to evaluate the usability, acceptability, and preliminary effectiveness of CARES as an assistive tool for guiding former family caregivers of people with dementia (n=3) in fidelity-adherent delivery to current family caregivers of people with dementia (n=12). The purpose of a field usability study is to assess the feasibility and acceptability of technology in users’ natural and conceptual environments [27]. Through field usability studies, researchers gain an understanding of the problems users encounter while using the system and gain insights into how individuals use the product [27]. The field usability study was conducted for 2 weeks to provide caregiver peer supporters and participants with the time to use and assess the CARES app and establish a peer connection. The field usability study was a first step in assessing the usability and acceptability of CARES, the study design, and the training. Caregiver peer supporters provided 5 to 7 hours of peer support per week, including video chats, messaging, and supervision. Each caregiver peer supporter was assigned 4 participants. In total, 17% (2/12) of the participants dropped out of the study due to a delay in the start date, and 8% (1/12) of the participants were excluded for not using the app and failing to initiate the field usability testing process, resulting in a final sample of 9 current family caregivers and 3 caregiver peer supporters. Study measures of burden, stress, and strain levels were administered via Qualtrics (Qualtrics International Inc) at baseline and at the end of the 2-week field usability study. Study measures on usability and acceptability were administered in an hour-long HIPAA-compliant videoconference semistructured interview at the end of the 2-week field usability study. All assessments were conducted by the PI.

### Ethical Considerations

The Committee for the Protection of Human Subjects at the Dartmouth Health Institutional Review Board approved the project (FP00002112). Participants provided their written informed consent. Participants were compensated for taking part in the study. Current family caregivers received US $20 for the baseline assessment, US $20 for the completion of the 2-week CARES field usability study, and US $20 for the completion of the semistructured interview conducted after the 2-week field usability study. Caregiver peer supporters received US $120 for the 6 hours of training, US $30 per hour for the 2-week field usability study, and US $30 for the completion of the semistructured interview conducted after the 2-week field usability study*.*

### Participants

A total of 12 participants were recruited from the Dartmouth-Hitchcock Aging Resource Center, memory cafés, and senior centers across New England and via dementia caregiver Facebook support groups to participate in the study with the goal of recruiting between 10 and 20 participants. Previous research has found that 10 users report approximately 80% of usability problems and 20 users report approximately 95% of usability problems for a given product [28]. The pilot study included 9 current family caregivers of people with dementia. Participants were eligible if they (1) were a current family caregiver of an individual with dementia, (2) were aged ≥18 years, (3) spoke and read English, and (4) provided voluntary informed consent for participation in the study. The study also included 3 former family caregivers of people with dementia. Participants were eligible if they (1) were a former family caregiver of an individual with dementia, (2) were aged ≥18 years, (3) spoke and read English, (4) were willing to use technology to deliver an intervention, and (5) provided voluntary informed consent for participation in the study. All participants were excluded if they (1) had a chart diagnosis of dementia or documented cognitive impairment as indicated by a Mini-Mental State Examination score of <24; (2) had major visual, hearing, or motor impairment; (3) had a terminal illness expected to result in death within 1 year; or (4) were patients with ≥2 emergency room visits or hospitalizations in the previous 6 months or determined by the clinical team to be psychiatrically or medically unstable.

### Measures

The usability of the CARES app was evaluated using the System Usability Scale (SUS). The SUS is a widely used, valid, reliable 10-item scale that assesses system usability [29]. Sample questions include “I think that I would like to use this system frequently” and “I thought the system was easy to use.” Response options range from 1 (*strongly disagree*) to 5 (*strongly agree*). Scores range from 0 to 100, with higher scores indicating better usability [30]. A mean SUS of ≥68 is indicative of an above-average user experience [30]. A system with an SUS score of >70 is considered acceptable. Scores of >85 are considered “excellent,” scores between 71 and 84 are considered “good,” and scores between 51 and 70 are considered “OK” [30].

Caregiver burden was assessed using the Zarit Burden Interview–Short Form. The Zarit Burden Interview–Short Form is a 12-item scale that evaluates caregivers’ physical burden, financial burden, interpersonal burden, and health [3]. The Zarit Burden Interview–Short Form is a valid scale for evaluating burden in caregivers of community-dwelling individuals with dementia [3]. Sample questions include “do you feel that because of the time you spend with your relative that you don’t have enough time for yourself” and “do you feel that you have lost control of your life since your relative’s illness.” Response options range from 0 (*never*) to 4 (*nearly always*). Scores range from 0 to 48, with higher scores indicating higher levels of burden.

The Modified Caregiver Strain Index is a 13-item scale that was used to assess strain and its consequences on caregivers’ overall health. The Modified Caregiver Strain Index is a stable and reliable measure of strain among long-term caregivers [7]. Sample questions include “caregiving is inconvenient” and “I feel completely overwhelmed.” Response options include “yes, on a regular basis,” “yes, sometimes,” and “no.” Scores range from 0 to 26, with higher scores indicating higher levels of strain.

Caregiver stress was assessed using the Caregiver Self-Assessment Questionnaire (CSAQ). The CSAQ is an 18-item scale that assesses the stress levels of family caregivers [31]. The CSAQ is a valid scale for individuals caring for people with dementia [31]. Sample questions include “during the past week or so, I have felt that I couldn’t leave my relative alone” and “during the past week or so, I have been satisfied with the support my family has given me.” Response options for questions 1 to 16 include “yes” or “no.” Caregivers are considered to have high levels of stress if they respond with “yes” to ≥10 questions. Question 17 asks the caregiver to rate their level of stress from “not stressful” to “extremely stressful” on a scale from 1 to 10. Caregivers are considered to have high levels of stress if they score ≥6 on question 17.

A semistructured interview was administered to assess the acceptability of the CARES app from the perspective of the participants. The interview questions were informed by the Consolidated Framework for Implementation Research (CFIR). The CFIR is a meta-theoretical framework that guides implementation research and is used to systematically assess potential barriers to and facilitators of implementing an intervention [32,33]. Previous studies on the feasibility of web-based tools and interventions have used the CFIR to guide qualitative analysis, identify aspects of implementation feasibility, and determine areas of improvement and adaptation to better meet the needs of users (eg, see the study by Lawson et al [34]). The PI used the CFIR Interview Guide Tool to develop the interview questions. Interview questions covered CFIR domains such as intervention characteristics (what key attributes of the intervention influence the success of implementation), patients’ needs and resources (the extent to which patient needs and barriers to and facilitators of meeting those needs are accurately known), implementation climate (shared receptivity of involved individuals to an intervention and the extent to which the use of that intervention will be supported), self-efficacy (individuals’ beliefs in their own capacity to successfully implement the intervention), and evidence strength and quality (individuals’ perceptions of the quality and validity of the intervention) [32]. Interview guide questions included the following: “what would you change about the CARES system and intervention?” “How well does the intervention align with your values and norms?” “What barriers will family caregivers of people with dementia face to delivering or participating in the intervention?”

### Procedures

#### Recruitment

The PI (CCP) met with staff members at the Aging Resource Center to discuss the purpose of the study and the recruitment process. Together, they identified potential groups within and outside the Dartmouth-Hitchcock Aging Resource Center to recruit both former family caregivers of people with dementia to be trained in the delivery of the CARES app and current family caregivers of people with dementia to receive the CARES intervention. If the potential participants met the inclusion criteria, they were contacted via email and provided with a description of the study. Participants were told that the study was for an honors psychology thesis that aimed to assess the usability, acceptability, and preliminary effectiveness of a technology-based and caregiver-delivered peer support program, CARES, intended to help current family caregivers of people with dementia manage burden, strain, and stress levels. If they were interested in the study, they agreed to meet with the PI via HIPAA-compliant videoconferencing software or telephone for a baseline interview. The baseline interview lasted approximately 20 to 60 minutes. For the baseline interview, the PI read through the informed consent forms and answered participants’ questions regarding the content of the study. After the baseline interview, participants who provided informed consent for the study were sent a copy of their informed consent form; sent a link to a baseline survey on Qualtrics with questions on demographic information and their current burden, stress, and strain levels; and provided with information on how to download and log in to the CARES app.

#### Training

Once 3 former caregivers of people with dementia were recruited, they completed 6 hours of CARES training over 2 days through HIPAA-compliant videoconferencing software. The CARES training was adapted from the Digital Peer Support Certification [35]. Fortuna et al [35,36] found that a combination of educational training (the Digital Peer Support Certification) and management of the PeerTECH system increased peer support specialists’ capacity to use features of the digital peer support technology [35]. The training was based on adult learning theory and experiential learning theory. Adult learning theory suggests that adults learn best when they use past lived experiences and past developed skills and knowledge to enhance their learning process [37]. Experiential learning theory consists of four principles: (1) concrete experience, (2) observation and reflection, (3) abstract conceptualization, and (4) active experimentation [38]. In the CARES training, former caregivers were taught new skills; asked to reflect on and connect new knowledge and skills to past experiences and situations; and, finally, asked to practice the new skills they had learned. Techniques such as reinforcement, summation, and teach-back were used in the CARES training to promote the mastery of peer support skills [35].

The CARES training focused on instructing the former caregivers on the basic principles and competencies in the delivery of digital peer support and evidence-based practices to manage stress. The training included an overview of the following topics: peer support, health and aging, engaging older service users with technologies, teaching older adults how to use technology, life review, acceptance, mindfulness, coaching and making a plan of action, recognizing negative thoughts, the art and science of adult learning theory, and the role of family and caregivers in technology. Facilitated group discussions were paired with a PowerPoint (Microsoft Corp) presentation. The PowerPoint presentation was provided to all caregiver peer supporters at the end of the training. After the 6-hour training, participants who provided informed consent for the study were sent a copy of their informed consent form, a link to a baseline survey on Qualtrics with questions on demographic information, a copy of the caregiver peer support training, and information on how to download and log in to the CARES app. The PI was available for technological support from Monday to Saturday between the hours of 9 AM and 5 PM.

#### Fidelity

Over the course of the 2-week field usability study, a member of the research team tracked the CARES app messages between the caregiver peer supporters and assigned participants to evaluate whether the caregiver peer supporters were providing peer support in line with the training.

#### Informed Consent

Before the 2-week field usability study, the participant was provided with a description of the study, shown the CARES app, and read aloud the consent form word for word. Participants were evaluated according to the study criteria. If the participant was eligible and provided informed consent to take part in the study, they then completed the baseline survey on Qualtrics.

### Quantitative Analyses

Descriptive statistics were used to describe the demographic characteristics of the study sample and the results of the SUS. A paired-sample 2-tailed *t* test was conducted to assess the difference between the baseline and 2-week burden, stress, and strain level scores for statistical significance. All incomplete survey responses were excluded from the analyses. Descriptive statistics and analyses were computed using the SPSS software (version 26; IBM Corp).

### Qualitative Analyses

Interview data were analyzed using the rigorous and accelerated data reduction (RADar) technique. The RADar method helps streamline the process of qualitative data analysis through its ability to organize, reduce, and analyze data in user-friendly software packages such as Excel (Microsoft Corp) [39]. In accordance with the RADar methodology, the interview transcripts were formatted into an all-inclusive Excel spreadsheet. The Excel spreadsheet column headings included participant number, question, response, code, and notes. One researcher assigned codes to each response. A priori codes and themes related to the CFIR framework were identified. These codes included the acceptability of CARES, user needs and resources, intervention characteristics key to the success of the implementation, self-efficacy, quality and validity of the intervention, and receptivity of users. Codes were derived from the interview data by carefully reviewing the transcribed text. The all-inclusive data table was then reduced to include only content that answered the overarching research questions and was of primary interest to the analysis. The remaining text and codes were then organized into themes that applied the CFIR framework and were adjusted to best fit the content covered in the qualitative interviews. The percentage of each theme was determined by dividing the frequency with which the theme was present in the interview quotes by the total number of interview quotes.

## Results

### Overview

Table 1 presents the sociodemographic characteristics of the sample at baseline. The sample of current family caregivers (9/12, 75%; the participants) had a mean age of 67.3 (SD 15.1) years and was predominantly female (6/9, 67%) and White (9/9, 100%), and most of them (4/9, 44%) cared for a spouse. The sample of former family caregivers (3/12, 25%; the caregiver peer supporters) had a mean age of 68.3 (SD 11.0) years and was predominantly female (3/3, 100%) and White (3/3, 100%). One of the caregiver peer supporters had experience caring for a parent with dementia (1/3, 33%), one had experience caring for a spouse with dementia (1/3, 33%), and the other had experience caring for a sibling with dementia (1/3, 33%).

**Table 1:** Sociodemographic characteristics of study participants (N=12).

Characteristics			Participants (n=9)		Caregiver peer supporters (n=3)
**Sex, n (%)**					
	Male	3 (33)		0 (0)	
	Female	6 (67)		3 (100)	
Age (years), mean (SD; range)			67.3 (15.1; 42-87)		68.3 (11.0; 61-81)
Race (White), n (%)			9 (100)		3 (100)
**State, n (%)**					
	Connecticut	1 (11)		1 (33)	
	Florida	1 (11)		0 (0)	
	Massachusetts	2 (22)		1 (33)	
	New Hampshire	3 (33)		0 (0)	
	Vermont	2 (22)		1 (33)	
**Smartphone owner, n (%)**					
	Yes	9 (100)		3 (100)	
	No	0 (0)		0 (0)	
**Relation to relative with dementia, n (%)**					
	Child	3 (33)		1 (33)	
	Parent	1 (11)		0 (0)	
	Sibling	1 (11)		1 (33)	
	Spouse	4 (44)		1 (33)	

A total of 3 participants were excluded from the study. In total, 33% (1/3) of these participants did not complete the 2-week CARES app field usability study and interview session. A total of 67% (2/3) of these participants did not complete the 2-week CARES app due to a delay in the 2-week field usability study with one of the caregiver peer supporters. The caregiver peer supporter delayed the start of their 2-week field usability study because the CARES app was not functioning on their smartphone. The remaining 9 participants and all 3 caregiver peer supporters completed the CARES intervention.

### Usability of CARES

**Figure 6:**
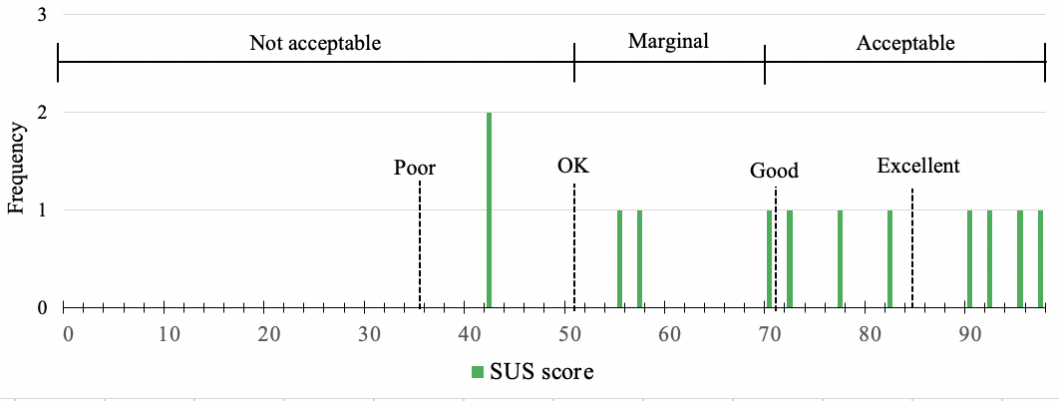
Distribution of results of the System Usability Scale (SUS) for study participants and interventionists.

Overall, participants reported above-average system usability on the SUS, with a mean score of 72.92 (SD 19.77) and a range from 42.50 to 97.50. Most participants found CARES to be an acceptable (8/12, 67%) and good or excellent (7/12, 58%) system. Specifically, the current family caregivers receiving support reported above-average system usability, with a mean score of 76.94 (SD 19.03) and a range from 42.50 to 97.50. Caregiver peer supporters reported marginal usability, with a mean score of 60.83 (SD 20.21) and a range from 42.50 to 82.50 on the SUS. One current caregiver and one caregiver peer supporter rated CARES as below OK. The distribution of the acceptability and adjective ratings as indicated by Bangor et al [30] are shown in Figure 6.

On average, participants sent 27 (SD 6.88) messages, with a range from 15 to 36, to their assigned caregiver peer over the course of the 2 weeks. All participants engaged weekly with the app. On average, participants reviewed 44% of the library resources over the course of the intervention, with a range from 0% to 100%.

Figure 6 shows the distribution of participant and interventionist (N*=*12) responses to the SUS. The x-axis marks the individual SUS scores, and the y-axis marks the frequency of the scores. Acceptability ranges and adjective ratings are informed by the work by Bangor et al [30].

### Barriers to and Acceptability and Facilitators of Using CARES

#### Overview

Regarding the acceptability of the CARES app and intervention, 24 codes and 6 themes related to the acceptance of CARES were identified. The 6 themes were improving the CARES app and intervention, acceptability of the CARES app features and design, value of the caregiver-peer relationship, barriers and limitations of CARES, caregiver needs and preferences, and caregiver challenges. The themes are listed in Table 2.

**Table 2:** Themes from qualitative analysis of the semistructured interviews.

Theme	Description	Participant quotes
Improving the CARES^a^ app and intervention	The participants and caregiver peer supporters provided input on how to improve the CARES app features and study protocol.	“If you’re dealing with some kind of messaging app...there’s no point if there’s no notification because nobody will think to go check it.” [participant 5] “One of my concerns was that a couple of the people didn’t understand what they were supposed to be doing, or how to interact with the app. I think that there needs to be a little bit more explanation upfront before we start interacting with [participants].” [caregiver peer supporter 14]
Acceptability of the CARES app features and design	Most participants and caregiver peer supporters found the CARES app to be acceptable for providing support to caregivers of individuals with dementia.	“I knew that right after, like the first couple of messages back and forth, I was like, this is a great idea. Because it’s convenient. It’s easy. It’s, you know, nonjudgmental. It’s just what you want from a support thing.” [participant 7] “I think that it’s like a personal support group. That’s what it is. And it’s in your pocket, because it’s on your phone and it’s delivered in an app, you don’t have to leave your home, you don’t have to try to arrange coverage to somebody to sit with, you know, your loved one, so that you can sneak out for an hour and then worry the whole hour that you’re out about what’s going on at home.” [caregiver peer supporter 13]
Value of the caregiver-peer relationship	Participants specifically highlighted their appreciation of the caregiver-peer relationship.	“You really felt like you had somebody to reach out to in the times when things were really stressful and really felt overwhelming. It just was somebody that you could connect with that knew how you were feeling.” [participant 7] “...knowing that there’s somebody out there who is thinking about me and my situation.” [participant 10]
Barriers and limitations of CARES	This refers to challenges that users face in using the CARES app and delivering the CARES intervention.	“The only barrier I see is, if somebody doesn’t have access to an iPhone.” [participant 8] “I’m so busy doing the caregiver stuff, and all the other sort of managing, so that if I have any time to myself, I would want to be doing other things that, you know, that didn’t involve caregiving. So I wouldn’t be apt to wanting to take the time out of those personal times.” [participant 10]
Caregiver needs and preferences	Most participants and caregiver peer supporters found that the CARES app met the needs and preferences of family caregivers of people with dementia.	“I think that a lot of caregivers will love [CARES] too. You know, the doctors are doctors, they’re doing the medical part of it. They don’t even think about the emotional part that the caregiver is going through.” [caregiver peer supporter 14] “It’s more I know that I have to take care of myself in order to be a better caregiver. And I can’t do that if I’m not feeling good about myself. And yet, I didn’t know how to do that...So I think I think [my peer supporter] was really good the way that she, she validated my feelings and, and was out there for me.” [participant 6]
Caregiver challenges	Participants highlighted the overall challenges of caring for a family member with dementia.	“My daughters were very concerned about me not getting out enough on my own.” [participant 9] “One of the biggest struggles that I have is finding people that understand what I’m going through...it’s very difficult to find somebody that I could connect with that had been through what I was going through, and that I felt comfortable really voicing my feelings to. So I think the idea behind the app is like, great, actually because, I mean, it’s like somebody that’s always there that knows exactly what you’re feeling and what you’ve been through.” [participant 7]

^a^CARES: Caregiver Remote Education and Support.

#### Improving the CARES App and Intervention

The most prevalent theme was improving the CARES app and intervention. This theme comprised 3 subthemes: improving the study, improving the app features, and technological difficulties. Improving the CARES app and intervention constituted 26% of the themes discussed in the interviews. The participants and caregiver peer supporters provided input on how to improve the CARES app features and study protocol. For example, a few participants noted problems receiving notifications when their assigned peer sent them a message through the app. Participant 5 mentioned the following:

...if you’re dealing with some kind of messaging app...there’s no point if there’s no notification because nobody will think to go check it.

Some participants found aspects of the app difficult to maneuver. At times, the video chat would not connect properly, and the library resources would appear blank. Caregiver peer supporter 13 thought that the home page of the CARES app should clarify the content of the library resource feature to increase interaction with the materials contained within the library:

...you don’t know that the resources are there, they’re offering them to you, but you can’t find them. It’s like you’re going on a board game, and you don’t know where to get off.

Other participants provided ideas on how to enhance the *app*. For example, caregiver peer supporter 15 suggested that the app include the option to communicate via telephone along with video chat and messaging as building rapport can be “very difficult to do with texting.” Other participants suggested adding a support group–like feature where participants could connect with multiple peers rather than just 1. Participant 5 suggested the following:

...having kind of a group chat or a message board, where you could just be like, okay, just, you know, like, venting for a second or whatever, that could be really kind of an expansion from beyond just the one on one.

The caregiver peer supporters also provided feedback on how to improve the peer support training. The caregiver peer supporters thought that clarification of the peer supporter and participant roles and expectations would have improved the participant-peer interaction and relationship. For example, caregiver peer supporter 14 stated the following:

...one of my concerns was that a couple of the people didn’t understand what they were supposed to be doing, or how to interact with the app. I think that there needs to be a little bit more explanation upfront before we start interacting with

participants

Both participants and caregiver peer supporters recommended holding a training specifically on the features of the CARES app. Caregiver peer supporter 13 said the following:

I think that it would have also been beneficial to download the app, and then on part of the training, you walk through it with us, and we...just play with it...like hands on learning.

The caregiver peer supporters suggested that the training should include additional practice using the CARES app and suggestions on how to initiate relationships with their assigned participants. Participants proposed that, in the future, a researcher should explain the features available on the CARES app and clarify their expectations for both the caregiver peer and the participant. Finally, participants recommended that future studies should match peers with participants based on their relationship to the individual with dementia they are caring for.

#### Acceptability of the CARES App Features and Design

The second theme, acceptability of the CARES *app* features and design, constituted 25% of the themes discussed in the interviews. Overall, most participants and caregiver peer supporters found the CARES app to be acceptable for providing support to caregivers of individuals with dementia. All participants agreed that the main purpose of the app was to connect caregivers with peers with a similar lived experience. Most participants interacted most with the messaging feature. Participant 5 mentioned the following:

I think the main point, or the main feature, is the connection to peers.

Participants and caregiver peer supporters emphasized the convenience and accessibility of the CARES app. For example, participant 7 said the following:

I knew that right after, like the first couple of messages back and forth, I was like, this is a great idea. Because it’s convenient. It’s easy. It’s, you know, non-judgmental. It’s just what you want from a support thing. And it’s also sort of on your own terms, though, because you get a message. So like, if you didn’t want to respond instantly, you can kind of gather your thoughts, and you have time to respond, unlike a regular back and forth support group where if somebody asked me a question, I kind of have to have an answer...So. I think this was better, because I had a few minutes to really think through what she was asking me...and I had a minute to kind of gather my thoughts, and then I could just type it back.

Participant 5 mentioned that, in contrast to in-person support groups where caregivers may “struggle with getting away even for an hour out of an apartment or their house for an hour,” they “like the fact that they can [use CARES] over an iPad or an iPhone...I think that definitely makes it more accessible and easy.” Caregiver peer supporters agreed that the CARES app was an appropriate tool for current caregivers of people with dementia. Caregiver peer supporter 13 shared the following:

I think that it’s like a personal support group. That’s what it is. And it’s in your pocket, because it’s on your phone and it’s delivered in an app, you don’t have to leave your home, you don’t have to try to arrange coverage to somebody to sit with, you know, your loved one, so that you can sneak out for an hour and then worry the whole hour that you’re out about what’s going on at home.

However, while all participants found the messaging feature of the CARES app beneficial, only some of the participants used the library resources (specifically the mindfulness, values, and negative thinking information). The “goals” and “wellness” features of the app were the least used by participants.

#### Value of the Caregiver-Peer Relationship

The third theme, the value of the caregiver-peer relationship, constituted 15% of the themes discussed in the interviews. Participants specifically highlighted their appreciation of the caregiver-peer relationship. Most participants emphasized that the purpose of the CARES app was the caregiver-peer connection. Participants found that their assigned caregiver peer supporters were knowledgeable, understanding, and validating. Participant 7 mentioned the following:

...you really felt like you had somebody to reach out to in the times when things were really stressful and really felt overwhelming. It just was somebody that you could connect with that knew how you were feeling.

They also shared the following:

...it was also nice, because the person that I was connecting with chose to be connected with somebody. So it wasn’t like you felt like you were burdening somebody else with your feelings.

The caregiver peer supporters also found value in the caregiver-peer relationship. Caregiver peer supporter 13 said the following:

I think it’s a great way to connect with caregivers. And it’s easy because you can just type a message and somebody picks up that message at that point, and it’s like having a support system at your fingertips...So I think when it works correctly, it would be a great effective tool for caregivers...because I found that caregivers in general, you know, they’re not healthcare professionals. And they’re expected to be in that role as part of the healthcare team. And they don’t even know like, who to call, like, do I call you when my husband is running a fever and needs care?...I feel for these people that are just like plunked down in this role. And they don’t have anyone. Even [one participant] said, I feel great, just knowing that you’re there. And you provided this emotional support for me. And it’s nice to know that you can reach out to someone that gets it. And that’s exactly what it is...You know, it’s a lifeline for people.

Participants and caregivers found that participants appreciated the intervention “knowing that there’s somebody out there who is thinking about me and my situation” (participant 10). The caregiver-peer connection was central to the CARES intervention.

#### Barriers and Limitations of CARES

The fourth theme, barriers and limitations of CARES, constituted 12% of the themes discussed in the interviews. Participants highlighted 2 main barriers and limitations: access to technology and time constraints. First, participants recognized that some caregivers may not have access to a smartphone or tablet or may not have adequate internet connection. Participant 8 mentioned that “the only barrier I see is, if somebody doesn’t have access to an iPhone.” Other participants noted that older adults may not be comfortable using technology. Time constraints and competing priorities were cited as other barriers that participants might face when using the CARES app. For example, participant 10 shared the following:

I’m so busy doing the caregiver stuff, and all the other sort of managing, so that if I have any time to myself, I would want to be doing other things that, you know, that didn’t involve caregiving. So I wouldn’t be apt to wanting to take the time out of those personal times.

Finally, many participants and caregiver peer supporters thought that a 2-week period did not provide enough time to fully explore the features of the CARES app and peer relationship. For example, participant 7 said that “well, I didn’t really check it out as much as I wanted to, because two weeks is not a lot of time.”

#### Caregiver Needs and Preferences

The fifth theme, caregiver needs and preferences, constituted 11% of the themes discussed in the interviews. Overall, most participants and caregiver peer supporters found that the CARES app met the needs and preferences of family caregivers of people with dementia. Specifically, participants found that the CARES app and intervention provided social and emotional support. Participant 6 shared the following:

I think sometimes it’s important for people who are caregivers to just be able to say how they’re, how they’re feeling.

Caregiver peer supporter 14 mentioned the following:

I think that a lot of caregivers will love [CARES] too. You know, the doctors are doctors, they’re doing the medical part of it. They don’t even think about the emotional part that the caregiver is going through.

For many participants, the CARES app and intervention provided individualized care and support. For example, participant 6 shared the following:

...it’s more I know that I have to take care of myself in order to be a better caregiver. And I can’t do that if I’m not feeling good about myself. And yet, I didn’t know how to do that. Because I’m so tied up, so wrapped up in this. So I think I think she was really good the way that she, she validated my feelings and, and was out there for me.

However, participants also stressed that caregiver peer supporters should have a general understanding of individuals’ backgrounds and access to resources when providing support. For example, participant 5 mentioned the following:

...there’s a wide range of resources that people have. I’ve seen this in the caregiver group I am a part of. Some people have planned well, and can afford help and support and some people don’t have that, and they have no family around...The support person...should have an awareness of saying...you need to just hire somebody to come in for an hour every day.

Caregiver peer supporters need to have an awareness of participants’ available resources and priorities when providing support. Finally, most caregiver peer supporters also felt that CARES met their needs and preferences as former caregivers. For example, caregiver peer supporter 13 shared the following:

I always feel purposeful when giving back. That’s most of the reason that I do coaching...I always feel a feeling of purpose. And there’s a lot of emotional support and a feeling of gratification that comes from giving that emotional support, because you have the lived experience that you can share with these other caregivers. And if you don’t have that experience, you don’t get it like you can be in that role, but you don’t truly get what they’re going through.

Caregiver peer supporters felt a sense of purpose while delivering the CARES intervention.

#### Caregiver Challenges

Finally, caregiver challenges was an emerging theme that constituted 5% of the themes discussed in the interviews. In their interviews, the participants highlighted the challenges of caring for a family member with dementia. Topics included difficulty taking time for oneself, frustration, anxiety about the unknown and upcoming changes, guilt, and the inability to find people who understand their situation. For example, participant 9 mentioned the following:

...my daughters were very concerned about me not getting out enough on my own.

Participant 6 said the following:

I just wish with this disease they could say, well, in six months you may experience this and another six months you may experience that.

Participant 2 stated the following:

...there are certain days you just feel more on top of things than others.

Some participants shared how the CARES app and intervention addressed their challenges:

But one of the biggest struggles that I have is finding people that understand what I’m going through. Because if you have not provided care for somebody with dementia, you really don’t understand like you can try to understand you can have the knowledge. But if you don’t have the experience, it’s very difficult to find somebody that I could connect with that had been through what I was going through, and that I felt comfortable really voicing my feelings to. So I think the idea behind the app is like, great, actually because, I mean, it’s like somebody that’s always there that knows exactly what you’re feeling and what you’ve been through.

participant 7

Others did not voice whether CARES attended to the specific challenges they faced as caregivers.

### Preliminary Effectiveness of CARES

Participants’ decreases in burden, strain, and stress levels were not significant. However, we were not powered to find significance; rather, the purpose of this study was feasibility and acceptability. Non–statistically significant improvements were observed in all measures. The results of the baseline and 2-week posttreatment assessment for the 9 participants who completed the CARES intervention are shown in Table 3.

**Table 3:** Changes in outcomes from before to after the field usability study (2 weeks) for study participants (N=9)^a^.

Measure	Participants, n (%)	Pretest assessment, mean (SD)	Posttest assessment, mean (SD)	*t* test (*df*)	*P* value	Effect size (Cohen *d*)
ZBI-12^b^	9 (100)	22.44 (10.04)	21.44 (11.34)	1.25 (8)	.25	0.42
MCSI^c^	9 (100)	11.22 (6.62)	11.00 (7.44)	0.41 (8)	.70	0.13
CSAQ^d^ total	9 (100)	6.56 (3.97)	5.78 (4.12)	1.31 (8)	.23	0.44
CSAQ stress	9 (100)	5.28 (2.71)	4.89 (2.84)	1.00 (8)	.35	0.33

^a^A 2-tailed paired *t* test was used to assess statistical significance.

^b^ZBI-12: Zarit Burden Interview–Short Form.

^c^MCSI: Modified Caregiver Strain Index.

^d^CSAQ: Caregiver Self-Assessment Questionnaire. The CSAQ total is the average number of “yes” responses on the CSAQ, and the CSAQ stress score is the mean score on question 17 of the CSAQ, which asked participants to rate their level of stress on a scale from 1 to 10.

## Discussion

### Principal Findings

The purpose of this study was to evaluate the usability, acceptability, and preliminary effectiveness of the CARES app and intervention. The pilot study demonstrated that a 2-week peer-delivered and technology-supported mental health intervention (CARES) was acceptable for both former family caregivers of people with dementia who delivered peer support and current family caregivers of people with dementia who received peer support. Current caregivers reported above-average usability of CARES, and former caregivers reported marginal usability. The pilot study demonstrated that it is possible to train former caregivers in peer support and the delivery of CARES and integrated psychoeducation and mental health interventions using technology. CARES was associated with non–statistically significant improvements in burden, stress, and strain levels.

The usability of the CARES app was demonstrated using the SUS. Most caregivers found the CARES app to be an acceptable and good system with above-average usability. The CARES app allowed peer caregivers to provide individualized support and provided caregivers with access to evidence-based mental health resources on topics such as mindfulness and acceptance. The usability of CARES was also demonstrated through participants’ capacity to use the smartphone and tablet app, completion of library resources on the app, and use of the messaging chat feature. Overall, most participants and caregiver peer supporters agreed that the CARES app and intervention were an acceptable tool to support family caregivers of people with dementia. However, participants and caregiver peer supporters identified areas in which the usability and acceptability of the app could be improved, and the caregiver peer supporters specifically reported marginal usability of the CARES app. Caregiver peer supporters may have reported below-average usability because of latency in messaging and other technological difficulties using the app. Future studies should examine the cause of differences in usability scores between current and former family caregivers and update the CARES app accordingly.

Participants suggested that improvement of technological features would strengthen the app’s ability to achieve its purpose of connecting caregivers with peers. Some participants and peers faced technological difficulties while using the app. For example, at times, the app would not notify participants of new messages. This created a barrier in participants’ and peers’ ability to communicate efficiently and effectively. Participants also provided suggestions on how to improve the app features. For example, participants suggested adding a telephone feature to the app. Participants believed that adding a telephone feature would allow caregivers to communicate via the medium of their preference and, therefore, increase their comfort level with technology and the caregiver-peer relationship. Participants also suggested adding a feature through which they could connect with more than one caregiver with a similar lived experience and suggested adding more caregiver-specific resources to the library resource page.

Despite technological limitations, most participants and peers found that the CARES app was an acceptable support intervention for family caregivers of people with dementia. Participants identified the caregiver-peer connection as the principal feature of the CARES app. Participants labeled the message-based support as convenient, easy-to-use, accessible, and individualized. The caregiver peer supporters were described as knowledgeable, understanding, validating, and supporting. Participants felt that the shared lived experience offered by the former caregivers better matched their needs and preferences for emotional and social support compared to professional medical and health care workers. On the other hand, the former family caregivers felt a sense of purpose and gratification while delivering the CARES intervention.

These findings suggest that technology- and peer-based interventions are usable and acceptable to family caregivers of people with dementia and that a smartphone app is a promising tool to support the mental and emotional health and well-being of family caregivers outside of an in-person or clinical setting. Task shifting informal caregiver digital mental health services to community members with lived experience has the potential to provide acceptable mental health interventions to family caregivers of people with dementia while addressing the current barriers and challenges with respect to accessing support. While it is estimated that nearly 153 million older adults will have dementia worldwide by 2050, mental health services for caregivers and their family members are limited due to an insufficient number of adequately trained geriatric mental health care providers [16,40]. Peer-delivered and technology-supported interventions have the potential to provide mental health services to family caregivers of people with dementia that are easily accessible and effective [36]. While caregiver psychosocial interventions have faced limitations due to time commitments, geographical location, requirements to meet in person, and failure to address the individualized needs of the caregiver, former and current family caregivers of people with dementia reported that the CARES app and intervention addressed the unique needs and experiences of consumers.

The results of the study support the hypothesis that former family caregivers of people with dementia have the knowledge and skillset to deliver trained peer support to current family caregivers. In previous studies, peers have been reported to be particularly effective at engaging participants in interventions. By sharing a lived experience, peers have the ability to develop alliances with participants and are viewed as more credible than traditional clinicians and providers [21]. With the use of technology-based messaging and support, participants were able to provide individualized support to caregivers at any time and location.

However, there are barriers and limitations to consider when using and delivering the CARES app and intervention. First, the CARES app is not accessible to caregivers who do not have a smartphone or adequate internet connection. In addition, time constraints may limit caregivers’ ability to interact with the app and their assigned peer. Offering peer support to family caregivers of people with dementia may place stress on the interventionists. Caregiver peer supporters involved in the study should be offered mental health support while delivering the intervention. Finally, participants and peer supporters suggested that the abrupt ending of the 2-week field usability study may leave caregivers without the resources and support systems they came to rely on to manage well-being. Future studies should provide caregiver participants with additional caregiver support resources at the end of the CARES field usability study.

This study is not without limitations. First, one member of the research and intervention development team conducted a qualitative analysis of the interview data and identified codes and themes, which may have biased the results. In addition, member checking was not used to validate the findings and assess the accuracy of the qualitative results. Second, caregivers’ input was not included in the initial development of the CARES app. Stakeholder engagement in the early stages of intervention development is essential to ensure that the modality and components are relevant to the community [41]. Future studies will incorporate caregivers’ feedback to further improve and adapt the CARES app and intervention. Third, some participants and caregiver peer supporters experienced technological difficulties with the CARES app. For example, 22% (2/9) of the participants and 33% (1/3) of the caregiver peer supporters were unable to receive notifications and, therefore, had delayed responses to messages. Another caregiver peer supporter experienced challenges sending messages and, consequently, had to delay the start of their 2-week field usability study. These technological difficulties could have impacted the results of the field usability study and SUS. Fourth, the participant response rate was not tracked during the recruitment process. Future studies should track the response rate to improve recruitment procedures and decrease bias (eg, nonresponse bias). Fifth, data on the frequency of use of CARES app features such as video chat, goal setting, and wellness plans were not tracked across participants. This information will be helpful for further understanding the acceptability and usability of CARES. Sixth, the caregiver peer supporters were not assessed regarding whether they had learned the topics presented in the training, and the competence of the training was not assessed. Therefore, it is unknown whether the training sufficiently educated caregivers on the delivery of peer support. Seventh, the fidelity of caregiver peer support was not systematically evaluated through audio interactions; rather, message data determined high fidelity to the peer support model. Future research will assess message and audio interactions to determine fidelity to the caregiver peer support model of care through audio recordings on the app. Caregiver peer supporters’ deviation from the training may have biased the results. Eighth, the participant eligibility criteria were broad and included all family caregivers of individuals with dementia aged ≥18 years. Criteria such as whether the caregiver lived with their care recipient and the number of hours spent caring for their relative with dementia could impact the acceptability, usability, and effectiveness of the CARES app and intervention. Future studies should investigate the influence of differing caregiver roles and responsibilities on CARES. Finally, demographic information on factors such as education and income level was not collected, which may have affected the results and the perceived usability of the app.

While the aim of this study was to evaluate the usability of CARES and assess the acceptability and potential barriers to using the CARES technology, future studies would benefit from a larger sample size and a longer trial duration. In the qualitative interviews, participants shared that they wished they had a longer period to explore the app and the caregiver-peer relationship. While the length of the study and sample size were consistent with a field usability study, longer trials would allow participants to further assess the usability and acceptability of the app and whether it meets their needs and preferences as caregivers [42]. A longer trial would also more accurately reflect the length and fluctuation of the dementia caregiving experience. Future studies would also benefit from a randomized controlled design (eg, an intervention group with CARES and caregiver peer support vs a control group) and a control of covariables influencing the outcomes to evaluate the effectiveness of the CARES training, intervention, and app in reducing burden, strain, and stress in family caregivers of people with dementia. For example, future studies should examine the influence of age; relationship to the individual with dementia receiving care; stage of dementia; years providing care; severity of dementia symptoms; severity of the family caregiver’s stress, strain, and burden levels; and the use of other caregiver support treatments and interventions on the effectiveness of the CARES app.

Future studies should also assign caregivers to peers based on dementia diagnosis or relationship to the individual with dementia. Matching caregivers with peers based on shared caregiver lived experiences may moderate the effectiveness of the CARES intervention. Future research would also benefit from a more diverse group of participants. Recruitment procedures should focus on recruiting a sample of participants representative of the demographics of the greater caregiver population. This includes recruiting more caregivers of color and caregivers who identify as male or nonbinary. Finally, future work should address the benefit of caregiver peer support for the family caregivers both delivering and receiving the mental health intervention. Caregiver peer support may have bidirectional effects. The caregivers providing support may see improvements in their mental health and well-being along with those of the participants they are supporting. As indicated by the study interview data, caregiver peer supporters felt a sense of purpose while delivering the intervention. Future studies should further assess the potential bidirectional influence of the CARES app and intervention.

### Conclusions

This pilot study demonstrated that it is possible to train former family caregivers of people with dementia to use technology and deliver the CARES mental health intervention to current family caregivers of people with dementia. These findings provide preliminary evidence that a peer-delivered and technology-supported intervention designed to improve burden, stress, and strain levels is feasible and acceptable.

## Appendix

Multimedia Appendix 1 Images of the Caregiver Remote Education and Support app features and distribution of the System Usability Scale participant results.

## Abbreviations

ACT: acceptance and commitment therapy
CARES: Caregiver Remote Education and Support
CFIR: Consolidated Framework for Implementation Research
CSAQ: Caregiver Self-Assessment Questionnaire
HIPAA: Health Insurance Portability and Accountability Act
PI: principal investigator
RADar: rigorous and accelerated data reduction
SUS: System Usability Scale

## Data Availability

The data sets generated during and analyzed during this study are available from the corresponding author on reasonable request.
