# Supplementary material for: Usability, Acceptability, and Preliminary Effectiveness of a Peer-Delivered and Technology-Supported Mental Health Intervention for Family Caregivers of People With Dementia: Field Usability Study
Source: JMIR Hum Factors. 2024 May 27;11:e41202. doi: 10.2196/41202 (PMC11165281; doi:10.2196/41202)
Supplement: Multimedia Appendix 1 [file humanfactors_v11i1e41202_app1.docx]

**Appendix**

**Baseline Current Caregiver Peer Support Survey Introduction
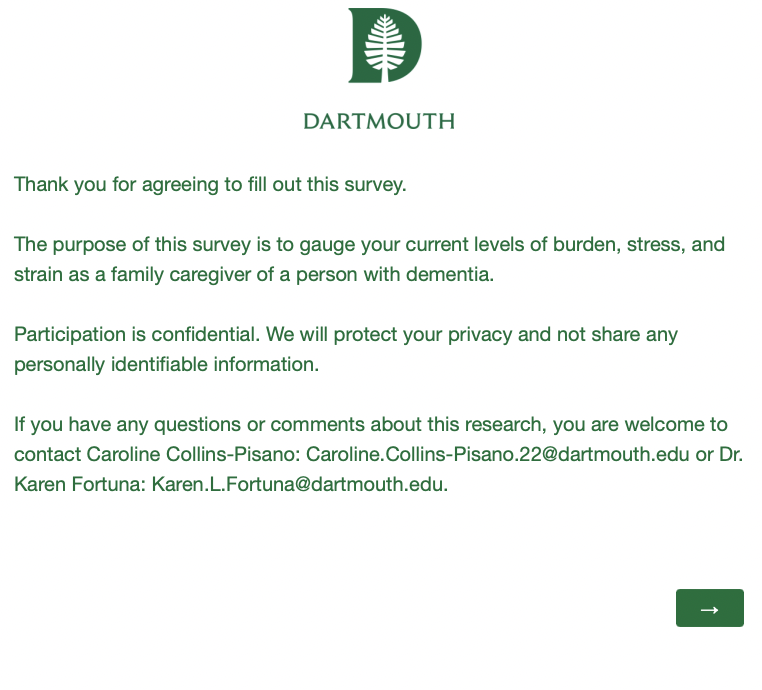
**

**“Short Form Zarit Burden Interview (ZBI-12)” Used in Pre and Post Survey**

**
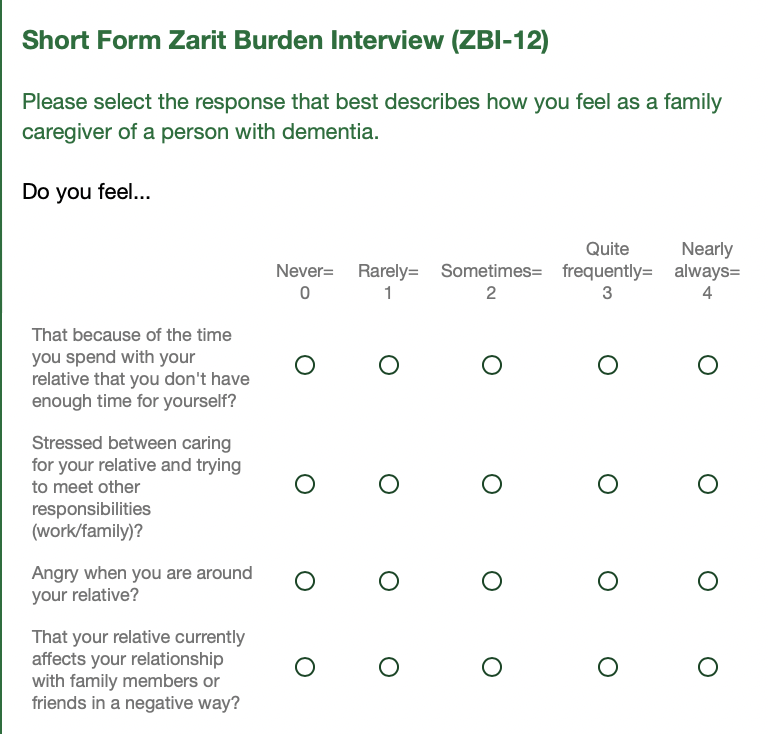
**

**
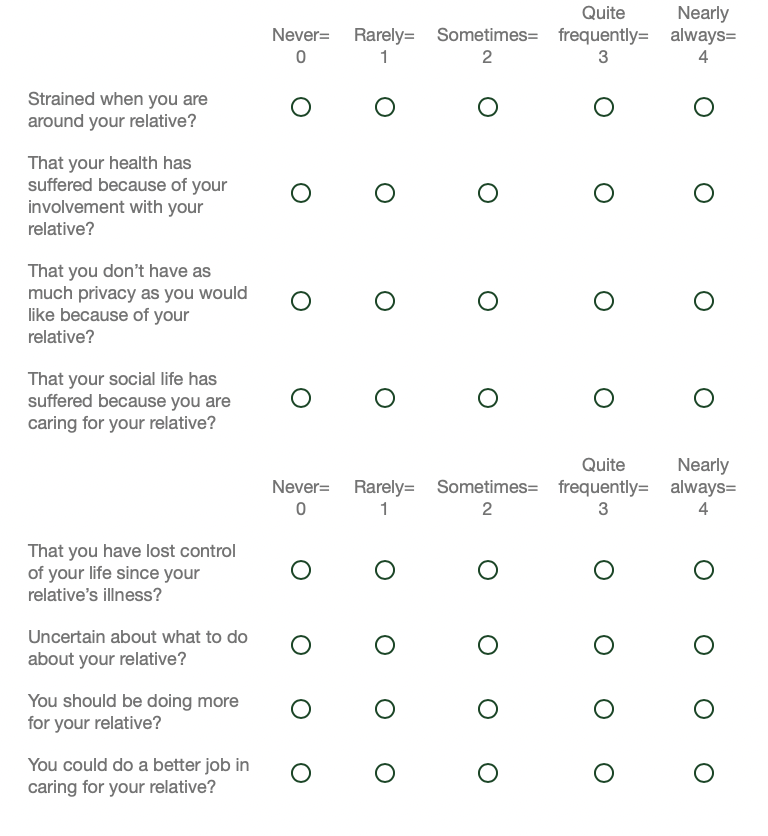
**

**“Modified Caregiver Strain Index” Used in Pre and Post Survey**

**
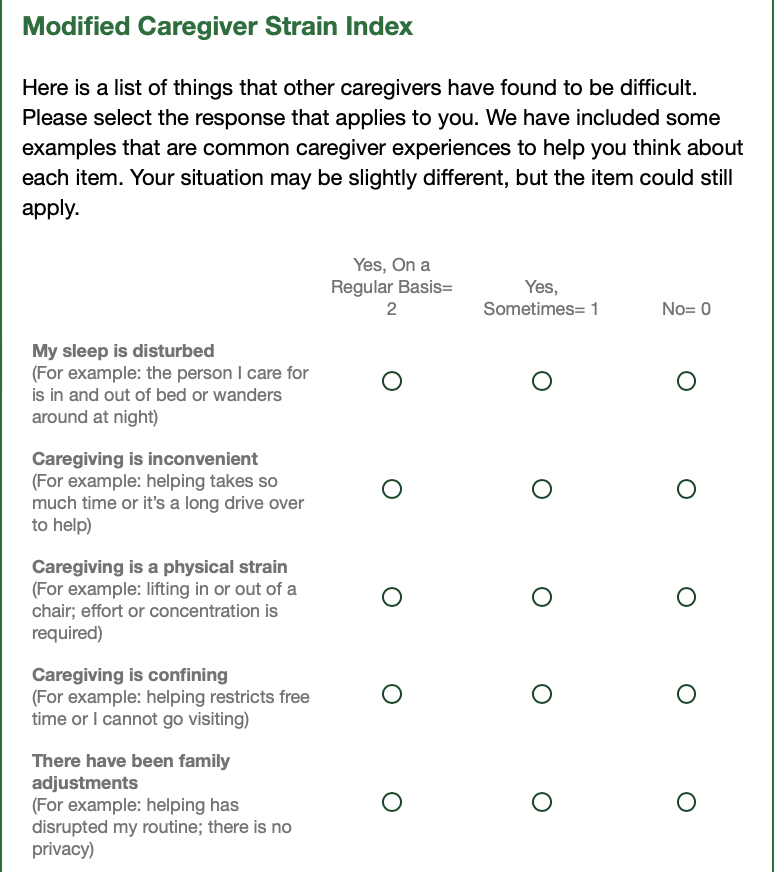
**

**
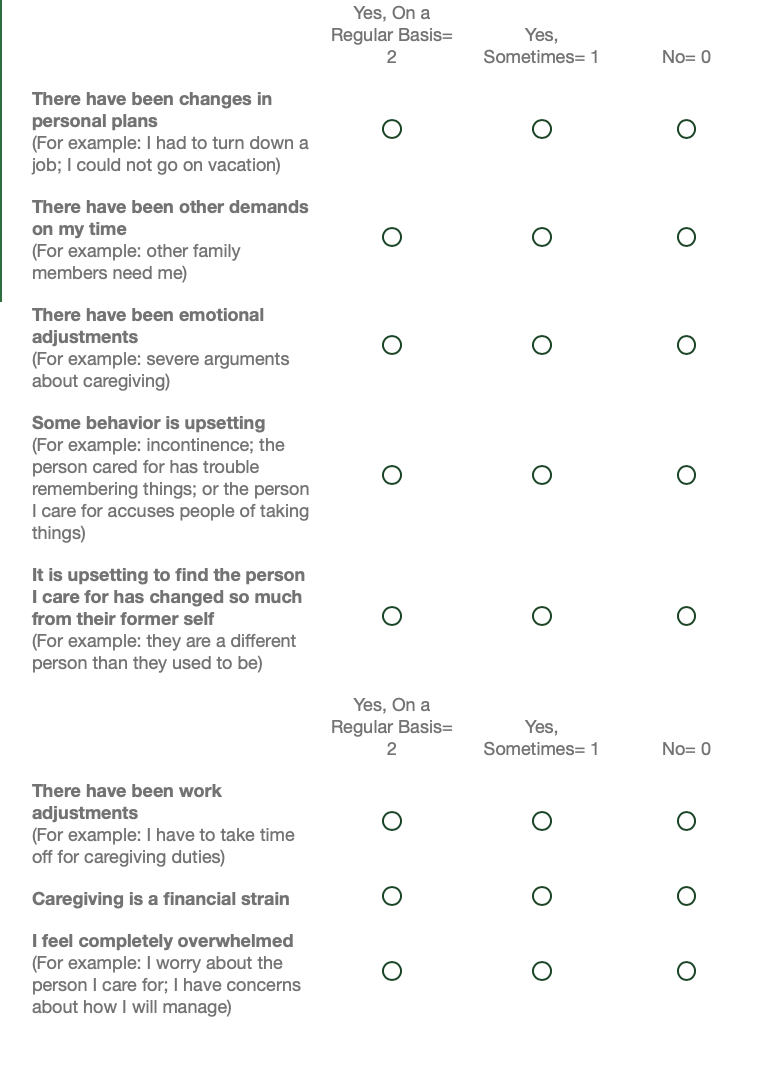
**

**“Caregiver Self-Assessment Questionnaire” Used in Pre and Post Survey
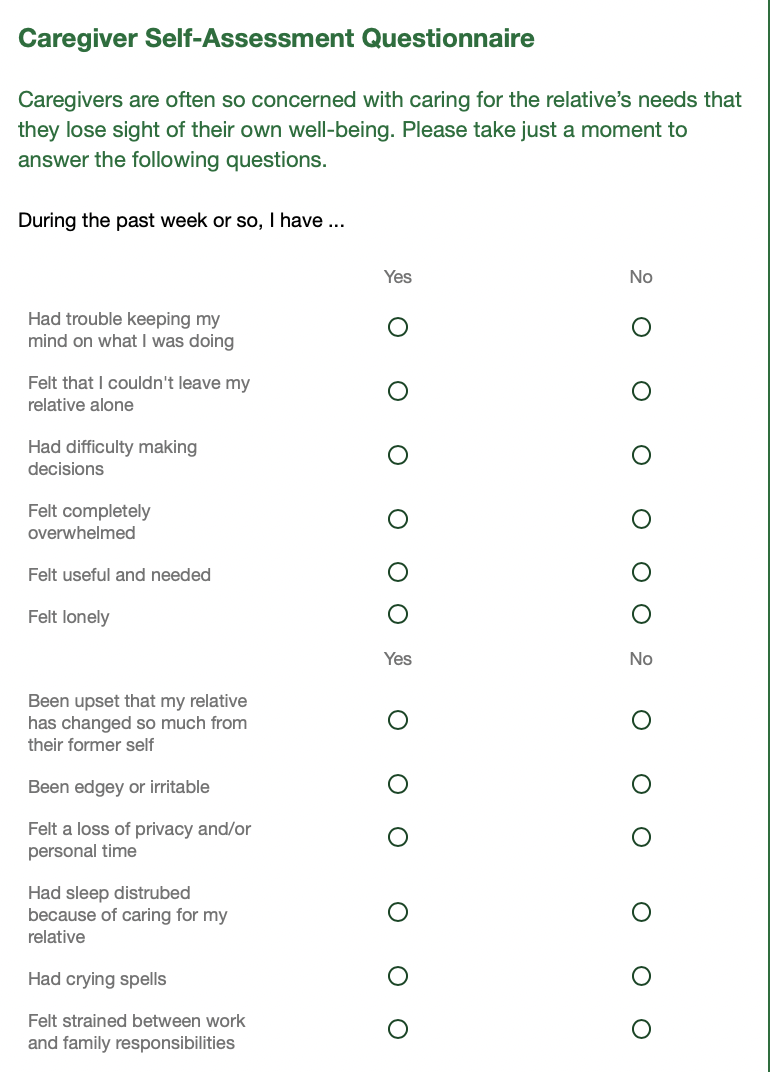
**

**
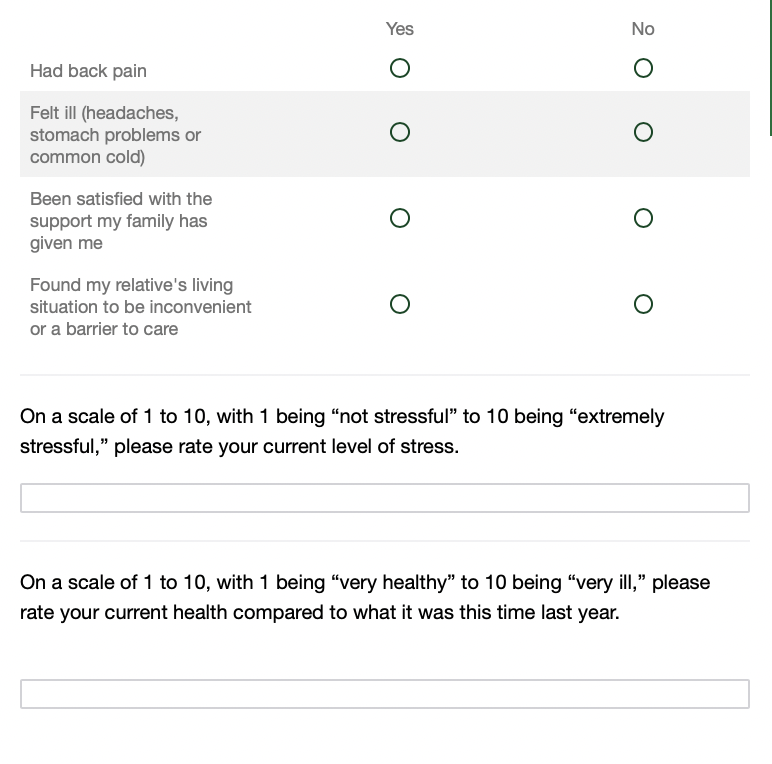
**

**Post-Trial Current Caregiver Peer Support Survey Introduction
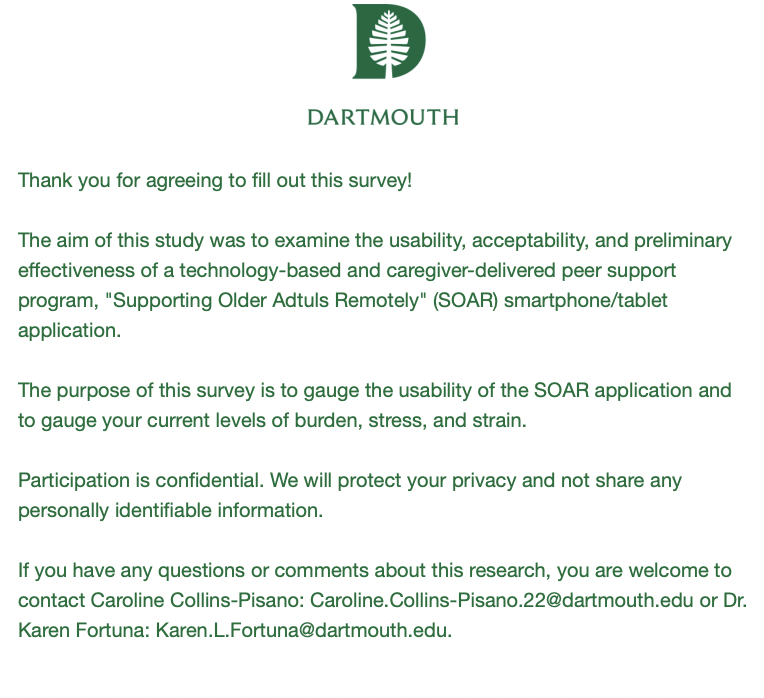
**

**“System Usability Scale” Used in Post Survey**

**
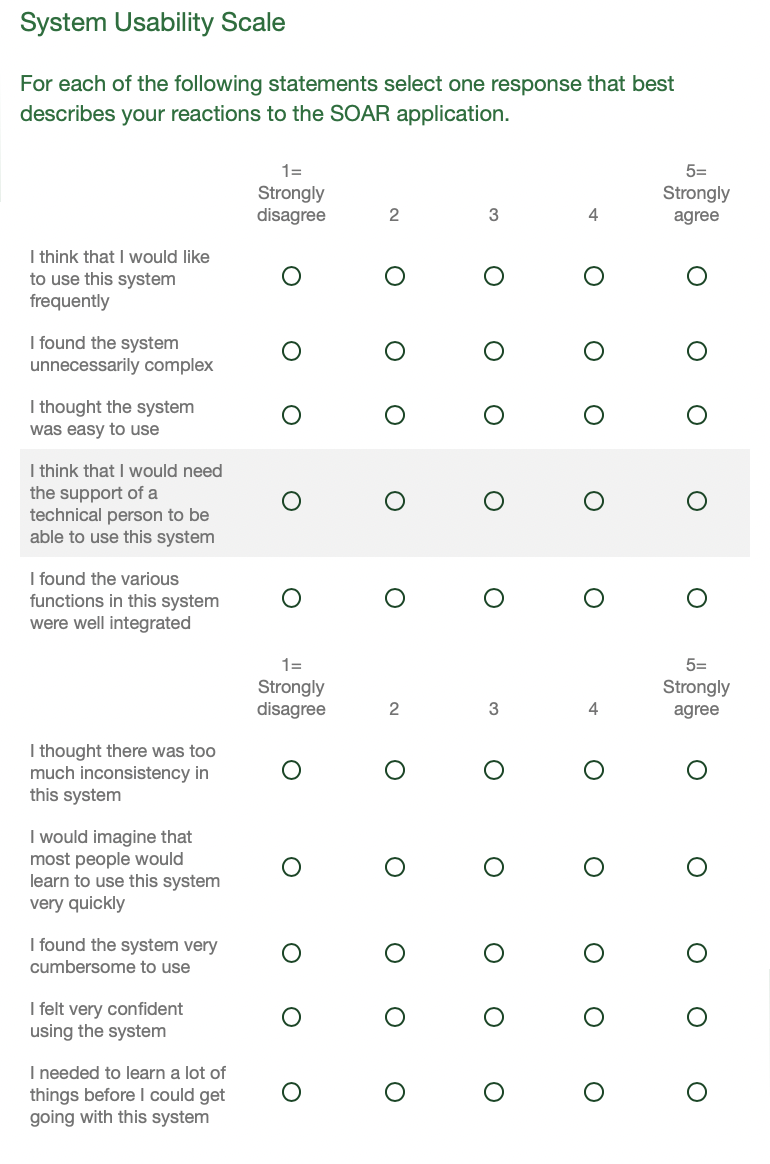
**

**“Digital Mental Health Peer Support Intervention for Family Caregivers of People with Dementia Acceptability and Ethical Barriers and Facilitators” Semi-Structured Interview Guide**

1. **Acceptability:** What do you like and dislike about the SOAR intervention (trainings, features, peer delivery)?
2. **Acceptability:** What would you change about the SOAR system and intervention (trainings, features, peer delivery)?
3. **Acceptability:** Do you feel that you benefited from the SOAR intervention (decreased stress, strain, and burden, feelings of purpose, social support, emotional support)?
4. **Acceptability:** Do you think the SOAR intervention achieved its purpose?
5. **Implementation Climate:** How well does the intervention align with your values and norms (does the intervention meet the needs and preferences of family caregivers of people with dementia)?
6. **Evidence Strength and Quality:** What kind of information or evidence are you aware of that shows whether or not the intervention will work in your setting (journal articles, word of mouth, low cost, etc.)?
7. **Patient Needs and Resources:** What barriers will the individuals served by your organization face to delivering or participating in the intervention (time commitment, access to resources, competing priorities, inadequate training, perceived lack of expertise, etc.)?
8. **Self-Efficacy:** How confident are you that you were able to carry out the actions and behaviors required to implement or participate in the intervention (comfort with technology, sufficient training)?
